# SNPFile – A software library and file format for large scale association mapping and population genetics studies

**DOI:** 10.1186/1471-2105-9-526

**Published:** 2008-12-08

**Authors:** Jesper Nielsen, Thomas Mailund

**Affiliations:** 1 Bioinformatics Research Center, University of Aarhus, Denmark; 2 Department of Computer Science, University of Aarhus, Denmark

## Abstract

**Background:**

High-throughput genotyping technology has enabled cost effective typing of thousands of individuals in hundred of thousands of markers for use in genome wide studies. This vast improvement in data acquisition technology makes it an informatics challenge to efficiently store and manipulate the data. While spreadsheets and at text files were adequate solutions earlier, the increased data size mandates more efficient solutions.

**Results:**

We describe a new binary file format for SNP data, together with a software library for file manipulation. The file format stores genotype data together with any kind of additional data, using a flexible serialisation mechanism. The format is designed to be IO efficient for the access patterns of most multi-locus analysis methods.

**Conclusion:**

The new file format has been very useful for our own studies where it has significantly reduced the informatics burden in keeping track of various secondary data, and where the memory and IO efficiency has greatly simplified analysis runs. A main limitation with the file format is that it is only supported by the very limited set of analysis tools developed in our own lab. This is somewhat alleviated by a scripting interfaces that makes it easy to write converters to and from the format.

## Background

High-throughput genotyping technology has enabled cost effective typing of thousands of individuals in hundred of thousands of markers for use in genome wide studies [1], in particular genome disease association studies [2-7].

There are currently no standard file format for storing such genotype data, and most major analysis tools define their own textual input and output formats. Only a few tools supports several input formats, and often several conversion scripts needs to be implemented in a study. These file formats of analysis tools usually only represent a restricted set of the data collected for the study – only the data necessary for the computations provided by the program – so a study either needs a secondary format for storing all data, with converter programs for import/export to analysis tools, or need several files for storing various types of data.

While spreadsheets and plain text files were adequate, if not optimal, solutions earlier, the increased data size mandates more efficient solutions. While plain text files formats have the advantage that they are human readable and can be edited in any text editor to correct mistakes, they have two major disadvantages: *i*) they are less space efficient than binary formats, often significantly so, and *ii*) text formats need to be parsed by tools before the data is analysed, a time consuming task when dealing with massive data sets.

Here we describe a new binary file format, *SNPFile*, for storing SNP data and a software library for manipulating such files. The file format stores genotype data together with any kind of additional data, using a flexible serialisation mechanism. Data is memory mapped as needed so even very large data sets can be manipulated with moderate RAM requirements. The representation is optimised for accessing nearby markers together, and thus cache and disk efficient for the access patterns of most multi-locus analysis methods.

We have extended the suite of association mapping tools developed in our group [8], including both single marker methods [9] and multi locus methods [10,11] and now successfully use it in our own studies.

## Results and discussion

We have developed a new file format and C++ library for manipulating SNP genotype data and arbitrary secondary data. The design allows us to store all genotype and secondary data in a single file, using a flexible serialisation framework. The genotype data representation is designed to be memory and IO efficient for the access patterns typical for multi-marker association mapping methods.

### Simple and efficient genotype data manipulation

The primary data in a SNPFile is genotype data, represented as a matrix with one or two rows per individual (depending on whether the phase of the genotypes is know or unknown) and one column per marker. A matrix representation for the primary data is a simple abstraction that makes it relatively easy to implement most analyses.

The actual implementation consists of a small hierarchy of classes for representing the data, depending on the size of the data and the usage pattern. Small matrices can efficiently be stored in RAM while for larger matrices we provide file storage. The abstraction for accessing the data is the same whether the actual data is stored in RAM or on disk.

**Figure 1:**
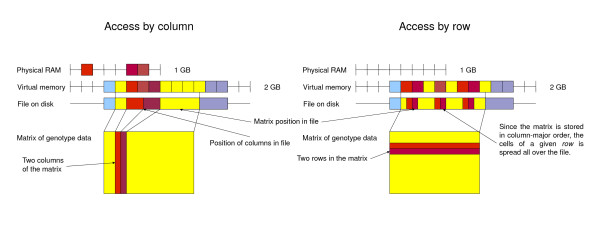
**Memory layout of SNPFile matrix**. If your program only accesses a few columns at a time they will cluster nicely in virtual memory and it will be easy for the operating system to keep only the needed pages in physical memory. This means you can handle very big SNPFiles while not using very much actual memory. Furthermore, if your program only access columns ordered left-to-right and from top to bottom, the file will simply be accessed from the beginning to the end. This is what the entire computer, both hardware and software, is optimized for. Thus it should be very fast. If you read a row from the matrix, however, you will access a lot of pages in the file, only use a very small part of each and the operating system will waste a lot of time reading data that will not be used, since it operates on entire pages.

**Figure 2:**
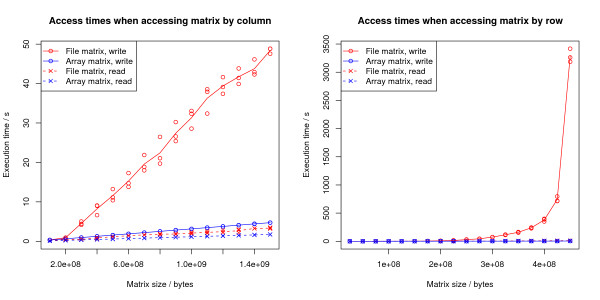
**Time for accessing matrices**. Times for reading or writing an entire matrix as a function of matrix size. Three tests was performed for each matrix size. The tests were done on a Intel Pentium 4 with 1 GB of RAM running linux.

Although the programming abstraction for RAM based and file based matrices are the same, the time performance can differ significantly between accessing RAM and file data. By representing the file based matrices in a "column by column" order on the disk (see figure 1) we have optimised the code for the common case where nearby markers (nearby columns in the matrix) are accessed together (see figure 2).

We do not expect the disk storage matrices to ever outperform RAM matrices. When the data can fit in RAM, the RAM matrices will always be more efficient to access, and when the matrix cannot fit in RAM, the swapping strategy will be similar to the swapping strategy used for the memory mapped file matrices. The main advantage of using the disk based matrices is that the matrix representation is directly available after opening the file; potentially time and memory consuming parsing is avoided.

Table 1 compares the running time of our *Blossoc *tool [12] when loading data into RAM from its previous flat text format with the running time when using SNPFile. *Blossoc *is a haplotype based association mapping tool. The algorithm with or without SNPFile is exactly the same, and the difference in running time is due to the parsing of the input data. For large input sizes, the text based version runs out of memory in the parsing routine, something that of course could be alleviated by more careful resource management. Using SNPFile, however, it is not necessary to worry about it.

**Table 1:** Runtime comparison using the Blossoc tool with its text file format vs. using SNPFile.

No. Individuals	Text IO	SNPFile
500	1200	867
1000	1893	1671

Running time in seconds, for Blossoc using text IO and SNPFile, as a function as the number of individuals.

### Framework for arbitrary secondary data

Depending on the analysis of the data, various secondary data is needed, such as phenotypes, co-variates etc. Most file formats support only a small fraction of the types of secondary data of interest in a study, since they only focus on the types of analysis intended when the file format was designed. A consequence is that data is often kept in several independent files, with ample risks for accidental inconsistencies between files.

To avoid such problems we have designed a flexible framework for secondary data into SNPFile. Through a serialisation framework, any C++ type can be stored in a SNPFile and accessed through a text key. Built-in types and STL containers are directly supported, and user-defined types can be supported by writing serialisation and de-serialisation methods. This can be done either through template methods in the user-defined types, or non-intrusively through global or name-space functions.

### Using SNPFile

Most multi-marker analysis methods can efficiently represent the genotype data in matrix form, with one or two rows (columns) per individual and with a column (row) per marker. For such methods, implementing them using SNPFile is straightforward. With the framework for storing arbitrary C++ data types, porting applications to use SNPFile is usually a simple matter of changing the IO routines to read the relevant secondary data through this framework, and then using SNPFiles matrix classes instead of those used before.

We have ported our existing association mapping software [8-11] – both single marker and multi-marker methods – to work on the new file format. Since these tools already represented genotype data as matrices, porting them was a simple task, taking from a few hours to a day or two. We are currently successfully using the updated tools in our own studies, where the format has greatly alleviated the informatics problems in data management and completely eliminated the need for cutting data into windows for analysis, when the full data cannot fit in RAM.

## Conclusion

The size of data that can cost efficiently be collected for population genetics studies – and especially disease mapping studies – has increased immensely the last few years, and this has lead to an informatics challenge in how to efficiently store and manipulate this data together with any secondary data collected for the study.

The file format we have described enables us to store all relevant data – primary and secondary – in a single file. The primary data is stored as a matrix, with a memory layout that makes it IO efficient to manipulate the data on disk, avoiding having to keep large data sets in RAM. The secondary data is stored using a flexible serialisation framework that allows any C++ data type to be stored together with the primary data.

The format has been very useful for our own studies where it has significantly reduced the informatics burden in keeping track of various secondary data, and where the memory and IO efficiency has greatly simplified analysis runs. A main limitation with the file format is that it is only supported by the very limited set of analysis tools developed in our own lab. Through scripting interfaces to the file format, we hope to alleviate this in the future.

A different binary file format for massive genotype data is available in the PLINK project [13]. The purpose of the binary format there is also achieving better CPU and memory performance. Where their format differs from ours is mainly in the treatment of secondary data. In the PLINK project, secondary data such as co-variates requires separate files from the genotype data. In contrast, we have designed our format such that we can store arbitrary secondary data together with the primary data in the same files.

## Methods

A SNPFile stores primary data as a matrix as well as any kind of secondary data, e.g. individuals phenotypes, marker names and positions, ethnicity of individuals or co-variates for disease studies.

### File manipulation

SNPFiles are accessed through a class of the same name. Instances of the class SNPFile represents a SNPFile on disk, and arguments to its constructor determine read, write and creation semantics of the file. The constructor for SNPFile looks like this:

SNPFile(const std::string &filename,

   bool allowWriting = false,

   bool createFile = true,

   int mode = 00644);

where filename specifies the name of the file on disk, allowWriting determines if the file should be opened read-only or in read-write mode, createFile specifies if the file should be created on disk if it does not already exist, and mode specifies the access file permissions. A usage example could look like this:

#include <snpfile/snpfile.hh>

using namespace BiRC::SNPFile;

int main( )

{

   SNPFile readOnly("/dir1/file1.snp");

   SNPFile readWrite("/dir2/file2.snp", true);

   // Do computations with files

   readOnly.close( );

   readWrite.close( );

   return 0;

}

The read/write semantics of a SNPFile is carried over to the methods and classes for accessing data, in the form of separate interfaces to mutable and immutable matrices, providing a compile time check for correct access to the data. The exception to this design is the SNPFile class itself: the access to SNPFile objects is checked at runtime, with exceptions thrown in case of incorrect access. The reason for this is to permit write access permission to change at runtime for interactive applications.

### Accessing genotype data

The primary data in a SNPFile is genotype data. We represent this as a matrix with each cell containing a genotype or allele. The matrix has one row for data with unknown phase and two rows for data with known phase. The matrix has one column per typed marker. The cells contain a signed char representing the genotype, where by convention, we use -9 to indicate missing values, use 0 and 1 for homozygote genotypes and 2 for heterzygote genotypes. Other values are reserved for future use.

The library contains a small hierarchy of matrix classes for representing genotype data, together with two handler classes providing the matrix interface to the data representation classes. The matrix data representation hierarchy, rooted in the abstract class MatrixData, implements the memory management strategies, including allocation, deallocation and resizing. The handler classes, ImmutableMatrix and Matrix, provides the interface for accessing and, in the case of Matrix, modifying the matrices. Splitting the matrix classes in two responsibilities, data representation and data access, combines flexibility in representation with efficient data access. We get a flexible design for representing data both in RAM and on disk with no virtual function overhead when accessing the data.

#### Accessing matrices

Access to matrix data is through one of the classes ImmutableMatrix and Matrix. ImmutableMatrix represents a read-only matrix, and Matrix represents a read-write matrix. The later is derived from the former, but allows entries in the matrix to be updated. A usage example, calculating the genotype frequencies for all markers in a matrix, is shown below:

#include <iostream>

#include <snpfile/matrix.hh>

using namespace BiRC::SNPFile;

void genotypeFrequencies(ImmutableMatrix &m)

{

   for (int j = 0; j < m.noCols( ); ++j) {

      int counts[ ] = {0,0,0};

      int total = 0;

      for (int i = 0; i < m.noRows( ); ++i) {

         if (m(i, j) < 0) continue; //missing

         if (m(i, j) > 2) continue; //error

         ++counts [m(i, j)];

         ++total;

      }

      if (total > 0) {

         std::cout << counts [0]/total << ' '

            << counts [1]/total << ' '

            << counts [2]/total

            << std::endl;

      } else {

         std::cout << "nan nan nan"

            << std::endl;

      }

   }

}

Both handler classes can only be instantiated when assigned a MatrixData instance. They do not represent the data but only provide interfaces to it.

#### Matrix representation

The MatrixData class is abstract and provides the bridge between data access (the ImmutableMatrix and Matrix classes) and memory management. The actual data management is implemented in sub-classes of MatrixData. The library provides two data representations, one for representing matrix data in RAM and one for representing data on disk (the later actually implemented as two different classes), but application programmers can provide their own as needed.

In our design we have considered the data representations implementation details, so the actual class representations cannot be accessed through the library interface. Instead, instances of the classes can be created through factory methods.

Small matrices, representing small windows of the data, are often used as part of a larger computation, and such matrices are most efficiently stored in RAM. The ArrayMatrixData class is provided for this. The factory method for creating instances of this class returns a Matrix handler since read-only RAM based matrices are of little use. This handler can, of course, always be cast to a ImmutableMatrix handler if a read-only interface is needed for later processing.

Matrices stored on disk are handled by the two classes: ReadOnlyFileMatrixData and ReadWriteFileMatrixData. Both are constructed with a reference to a SNPFile object. The factory method for ReadOnlyFileMatrixData returns a ImmutableMatrix instance while the factory method for ReadWriteFileMatrixData returns a Matrix. It is a runtime error to create a ReadWriteFileMatrixData object with a reference to a SNPFile object opened as read-only. Compile time checks ensure that the access patterns to matrices, after their instantiation, is correct.

An example of accessing a matrix on a read-only file, for calculating the genotype using the function defined above, is shown below:

#include <iostream>

#include <snpfile/snpfile.hh>

#include <snpfile/matrix.hh>

#include <snpfile/file_matrix.hh>

using namespace BiRC::SNPFile;

void genotypeFrequencies(ImmutableMatrix &m)

{

   // see above for implementation

   ...

}

int main( )

{

   SNPFile file("filename.snp");

   ImmutableMatrix m = newReadOnlyFileMatrix(file);

   genotypeFrequencies(m);

   file.close( );

   return 0;

}

#### IO efficiency for large genotype data sets

One of the motivations for SNPFiles is efficient management of large datasets; frequently datasets too large to keep in the computer's main memory. We achieve this by keeping file matrices (classes ReadOnlyFileMatrixData and ReadWriteFileMatrixData) on disk, rather than loading them into RAM, and then use memory mapping to access the matrices. By representing the matrices on disk in a way that matches common usage we can rely on the operating system of the computer to make sure the right parts of the file are read into memory and flushed back to disk in an efficient way. This essentially means representing the matrices column-wise since most multi locus methods access neighbouring markers together but less frequently distant markers together.

#### Matrix views

For computations on sub-matrices, where the matrix data is not modified, it is inefficient to copy the data. It is often also inconvenient to design the methods to keep track of relevant indices for sub-matrices, especially with recursive methods that modify the views – e.g. split rows based on genotypes or sort columns with respect to marker position as in the Blossoc method [12].

The MatrixView class provides a solution to this problem by wrapping MatrixData objects and modifying matrix indices so a cell index in a view is redirected to the corresponding cell index in the matrix. This design allow transparent rearrangement of rows and columns, and extraction of arbitrary sub-matrices, with very little computational overhead.

### Accessing secondary data (meta data)

SNPFiles can store arbitrary secondary data, or meta data, associated to the primary data. Meta data access is handled through three template methods:

template<typename T>

void getMetadata(const MetadataAccessor &acc,

   const std::string &key,

   T &dest);

template<typename T>

T fetchMetadata(const MetadataAccessor &acc,

   const std::string &key);

template<typename T>

void setMetadata(MetadataAccessor &acc,

   const std::string &key,

   const T &src);

The first parameter for all tree functions is of type MetadataAccessor. A MetadataAccessor is a container capable of storing meta data, which in practise is usually a SNPFile object. The second parameter is a key used to identify the data. Since we can store arbitrary meta data, keys are used to identify the various data.

The functions are templates parameterized with the type of the meta data. In principle, any C++ type can be used as meta data, but the template functions needs to know how to serialise data of the type. For serialisation, we use a framework similar to the the Boost serialisation framework [14], but one that is binary compatible across different platforms and different versions of the C++ STL. The framework can immediately serialise all primitive types, such as int or double, and the most common STL types, such as map<> or vector<>.

Adding meta data to a SNPFile is done using setMetaData as below:

#include <snpfile/metadata_access.hh>

#include <snpfile/snpfile.hh>

#include <iterator>

#include <fstream>

using namespace BiRC::SNPFile;

using namespace std;

namespace {

   // command line options...

   bool binaryPhenotypes;

   // ... more options ...

}

int main(int argc, char *argv[ ])

{

   // ... option parsing ...

   SNPFile file("someFile.snp", true);

   ifstream input("input.txt");

   if (binaryPhenotypes) {

      // read a sequence of binary phenotypes

      // into a boolean vector

      vector<bool> phenotypes;

      copy(istream_iterator<bool>(input),

         istream_iterator<bool>( ),

         back_inserter(phenotypes));

      // store a flag indicating the

      // phenotypes are binary

      setMetadata(file, "binary phenotypes?", true);

      // store the phenotypes as well

      setMetadata(file, "phenotypes", phenotypes);

} else {

      // read a sequence of quantitative

      // phenotypes into a double vector

      vector<double> phenotypes;

      copy(istream_iterator<double>(input),

         istream_iterator<double>( ),

         back_inserter(phenotypes));

      // store a flag indicating the

      // phenotypes are *not* binary

      setMetadata(file, "binary phenotypes?", false);

      // store the phenotypes

      setMetadata(file, "phenotypes", phenotypes);

   }

   file.close( );

   return 0;

}

where the example code reads some phenotype data from a text file – either binary traits or continous traits depending on command line options – and stores the data, together with a flag, in the SNPFile. Reading the data back from a file is done through getMetadata or fetchMetaData:

#include <snpfile/metadata_access.hh>

#include <snpfile/snpfile.hh>

using namespace BiRC::SNPFile;

using namespace std;

int main( )

{

   SNPFile file("someFile.snp");

   if (fetchMetadata<bool>(file, "binary phenotypes?")) {

      vector<bool> phenotypes;

      getMetadata(file, "phenotypes", phenotypes);

      // ... analyse data ...

   } else {

      vector<double> phenotypes;

      getMetadata(file, "phenotypes", phenotypes);

      // ... analyse data ...

   }

   file.close( );

   return 0;

}

where fetchMetadata is just syntactic sugar around getMetaData so we can access data without necessarily declaring a variable for it (as in the if statement above). For complex data, such as maps, getMetaData is more efficient than fetchMetadata.

#### Serialising user-defined types

User defined classes cannot immediately be serialised, but it is possible to extend the serialisation framework with arbitrary types in two ways: by implementing member functions in the class or struct to be serialised, or by implementing free functions in the namespace of the class. The former can be used for classes the application programmer is free to modify, while the later can be used when that is not an option.

The simplest way to add serialisation through member functions it to add a template method named serialize that can be used for both serialisation and de-serialisation, depending on its template instantiation. Alternatively, an overloaded serialize function can be used to handle serialisation and de-serialisation differently. A class that implements serialisation with member functions can then be exported to the SNPFile serialisation framework using the macro BIRC_SNPFILE_INTRUSIVE_SERIALIZATION.

For non-intrusive serialisation, the framework can use a serialize template function in the namespace of the class to be serialised and the macro BIRC_SNPFILE_NONINTRUSIVE_SERIALIZATION instead. If different methods are needed for serialisation and de-serialisation, one can implement methods load and save in a specialised SerializationTrait template. For details on this, we refer to the library documentation.

The example below illustrates serialisation of user-defined types. The example shows how, in a hypothetical study where a SNPFile combines individuals from different previous studies and from different populations, we can store population and study information from the previous studies, and associate this to each individual. The example defines three new types: IndivData for data associated with each genotyped individual, StudyData for data associated with each previous study where the genotype data is obtained from, and PopulationData for data associated with populations. For IndivData we use the member function approach to serialisation and for the other two classes we use the free function version.

#include <snpfile/metadata_access.hh>

#include <snpfile/snpfile.hh>

using namespace BiRC::SNPFile;

#include <string>

#include <vector>

#include <map>

using namespace std;

struct IndivData {

   string name;

   int studyID;

   int populationID;

   // member interface to

   // meta data serialisation

   template<class Archive>

   void serialize(Archive & ar)

   {

      ar | name;

      ar | studyID;

      ar | populationID;

   }

};

// macro needed to export the serialisation

// to the SNPFile framework

BIRC_SNPFILE_INTRUSIVE_SERIALIZATION(IndivData);

struct StudyData {

   string someRelevantData;

   string additionalData;

};

struct PopulationData {

   string popData;

};

// non-intrusive support for serialisation

template<class Archive>

void serialize(Archive & ar, StudyData &d)

{

   ar | d.someRelevantData;

   ar | d.additionalData;

}

template<class Archive>

void serialize(Archive & ar, PopulationData &d)

{

   ar | d.popData;

}

// macros needed to export

// serialisation

BIRC_SNPFILE_NONINTRUSIVE_SERIALIZATION(StudyData);

BIRC_SNPFILE_NONINTRUSIVE_SERIALIZATION(PopulationData);

int main( )

{

   // mapping from studyIDs to study data

   map<int, StudyData> studies;

   // mapping from populationIDs to population data

   map<int, PopulationData> populations;

   // data for each individual in the SNPFile, ordered

   // in the same order as the rows in the genotype

   // matrix

   vector<IndivData> individualsData;

   // ... fill in data for the maps and vector...

   SNPFile file("someFile.snp", true);

   setMetadata(file, "study data", studies);

   setMetadata(file, "population data", populations);

   setMetadata(file, "individuals data", individualsData);

   file.close( );

   return 0;

}

After the serialisation method is specified in this way, getMetaData, setMetadata and fetchMetadata can be used as for any other type. For more information about serialisation of custom types, we refer to the library documentation.

#### Meta-data type system

The serialisation mechanism for meta-data requires that the program accessing meta-data knows the type of the data before accessing it – a consequence of using a statically typed language such as C++. Unfortunately, this limits the general usability of the meta-data framework: tools operating on SNPFiles must all agree on the availability and type of meta-data to be able to manipulate it. This requires a protocol that application programs must follow if their programs should be able to operate on the same files.

Our initial design did rely on such a meta-data protocol, with agreed-upon types for the data used by our tool suite. Our experience with this convinced us, however, that this approach was less flexible than desired. This lead us to design a system for storing type information together with meta-data in SNPFiles, enabling us to dynamically extract meta-data information – availability and type of meta-data. With this design, programs can probe SNPFiles to get information about meta-data, and users can – when the tools support this – interactively access data to do their analyses.

The meta-data type system is non-intrusive in the sense that it does not affect the interface to storing meta-data described above. Any kind of meta-data can still be serialised into SNPFiles – using the functions above – and type information will automatically be stored together with the data whenever the type of the data is known by the SNPFile library (which includes primitive types and STL containers).

For custom meta-data – where the SNPFile library does not know the type – a mechanism similar to the serialisation framework allows the application program to provide type information to SNPFile. For example, to add support for the three custom types introduced in the example above, we would just add the following lines to our program:

BiRC_SNPFILE_EXPORT_TYPE(IndivData)

BiRC_SNPFILE_EXPORT_TYPE(StudyData)

BiRC_SNPFILE_EXPORT_TYPE(PopulationData)

The macros used for exporting types to the serialisation framework will by default add type support as well, however, so in our example this is not needed.

In our program above, the type associated with meta-data "individuals data" will then be std::vector< IndivData >, the type associated with "population data" will be std::map< int32_t, PopulationData > and the data associated with "study data" will be std::map< int32_t, StudyData >. Without specifying the type this way, the types would be stored as std::vector< unknown > and std::map< int32_t, unknown >, respectively.

### Script access to SNPFiles

For easier manipulation of SNPFile files, we provide a Python extension module. Through this module, the genotype matrices can be manipulated in ways similar to the C++ interface. Most common meta data types can be serialized and manipulated, but due to type differences between Python and C++ there are some limitations in the Python interface, including manipulation of custom data types.

## Availability and requirements

**Project name: **SNPFile

**Project home page: **

**Operating system(s): **Binary distributions available for Linux. Source code available for all unix-like platforms.

**Programming language: **C++

**Other requirements: **The boost library [15].

**License: **GNU GPL version 2.

**Any restrictions to use by non-academics: **None, besides those of the GPL license.

## Abbreviations

IO: Input/Output; RAM: Random Access Memory; SNP: Single nucleotide polymorphism; STL: Standard template library.

## Competing interests

The authors declare that they have no competing interests.

## Authors' contributions

TM conceived of the project. JN and TM both designed the library, while JN did the majority of the implementation. Both authors drafted the manuscript.
